# Antimicrobial resistance genetic factor identification from whole-genome sequence data using deep feature selection

**DOI:** 10.1186/s12859-019-3054-4

**Published:** 2019-12-24

**Authors:** Jinhong Shi, Yan Yan, Matthew G. Links, Longhai Li, Jo-Anne R. Dillon, Michael Horsch, Anthony Kusalik

**Affiliations:** 1 Department of Computer Science, University of Saskatchewan, 110 Science Place, Saskatoon, S7N 5C9 Canada; 2 Department of Animal & Poultry Science, University of Saskatchewan, 51 Campus Drive, Saskatoon, S7N 5A8 Canada; 3 Department of Mathematics and Statistics, University of Saskatchewan, 106 Wiggins Road, Saskatoon, S7N 5E6 Canada; 4 Department of Biochemistry, Microbiology and Immunology, University of Saskatchewan, 107 Wiggins Road, Saskatoon, S7N 5E5 Canada; 5 Vaccine and Infectious Disease Organization – International Vaccine Center, 120 Veterinary Rd, Saskatoon, S7N 5E3 Canada

**Keywords:** SNP, Antimicrobial resistance, Feature selection, Deep neural network, *Neisseria gonorrhoeae*

## Abstract

**Background:**

Antimicrobial resistance (AMR) is a major threat to global public health because it makes standard treatments ineffective and contributes to the spread of infections. It is important to understand AMR’s biological mechanisms for the development of new drugs and more rapid and accurate clinical diagnostics. The increasing availability of whole-genome SNP (single nucleotide polymorphism) information, obtained from whole-genome sequence data, along with AMR profiles provides an opportunity to use feature selection in machine learning to find AMR-associated mutations. This work describes the use of a supervised feature selection approach using deep neural networks to detect AMR-associated genetic factors from whole-genome SNP data.

**Results:**

The proposed method, DNP-AAP (deep neural pursuit – average activation potential), was tested on a *Neisseria gonorrhoeae* dataset with paired whole-genome sequence data and resistance profiles to five commonly used antibiotics including penicillin, tetracycline, azithromycin, ciprofloxacin, and cefixime. The results show that DNP-AAP can effectively identify known AMR-associated genes in *N. gonorrhoeae*, and also provide a list of candidate genomic features (SNPs) that might lead to the discovery of novel AMR determinants. Logistic regression classifiers were built with the identified SNPs and the prediction AUCs (area under the curve) for penicillin, tetracycline, azithromycin, ciprofloxacin, and cefixime were 0.974, 0.969, 0.949, 0.994, and 0.976, respectively.

**Conclusions:**

DNP-AAP can effectively identify known AMR-associated genes in *N. gonorrhoeae*. It also provides a list of candidate genes and intergenic regions that might lead to novel AMR factor discovery. More generally, DNP-AAP can be applied to AMR analysis of any bacterial species with genomic variants and phenotype data. It can serve as a useful screening tool for microbiologists to generate genetic candidates for further lab experiments.

**Electronic supplementary material:**

The online version of this article (10.1186/s12859-019-3054-4) contains supplementary material, which is available to authorized users.

## Background

Antimicrobial resistance (AMR) is a natural feature of microbial ecosystems. In a therapeutic context, AMR is the ability of a microorganism to stop a medication from working against it. AMR is a major threat to global public health because it makes standard treatments ineffective and contributes to the spread of microbial infections. It is estimated that 700,000 deaths were attributable to AMR in 2016 and that this number will increase to 10 million by 2050 if no actions are taken to tackle this problem [1]. One vital step in fighting AMR is identification of resistance determinants, such as single nucleotide polymorphisms (SNPs), from whole-genome sequence (WGS) data so that AMR’s biological mechanisms can be studied and understood. This understanding will provide crucial insights into the design and development of rapid and accurate clinical diagnostics for AMR as well as new antimicrobial drugs.

It is becoming increasingly feasible to predict AMR phenotypes directly from whole-genome SNP data as the cost of genotyping is continually decreasing with the advance of rapid and high-throughput sequencers. It is advantageous to predict AMR phenotypes from whole-genome genotype data because it does not require bacterial growth, pure cultures or previously identified marker genes as in vitro phenotype tests [2, 3]. In order to make such predictions, the variations between individual genomes are examined and related to phenotypes. To this end, a genome-wide association study (GWAS) is commonly performed to detect associations between SNPs and AMR phenotypes [4]. This is one way to address the curse of dimensionality—the feature dimension being much higher than the sample size—in building models to predict phenotypes from genotypes. A standard GWAS calculates a p-value for each SNP by performing a statistical significance test and sets a threshold to output only the most significant SNPs. The primary limitation of this approach is that the results are sensitive to the degree of match between the assumed statistical model and the real data distribution. One demonstration of this point is that different GWAS packages often output different detected SNPs and some with causal SNPs missing. Moreover, p-values from GWAS only indicate whether or not SNPs are related to a phenotype, but not how strongly they are related. This is one reason why SNPs selected by GWAS are not always good predictors, and why we cannot completely rely on them as features to build predictive models. In this regard, machine learning algorithms can serve as an alternative and complementary method to GWAS.

Machine-learning algorithms can identify relevant features in a complex dataset or make accurate predictions from such data. In the context of predicting AMR phenotypes based on whole-genome sequence (WGS) data, there are many examples of applying machine-learning methods to the problem [2, 3, 5–7]. For instance, a logistic regression classifier was implemented to classify the susceptibility phenotype consistent with vancomycin-intermediate *Staphylococcus aureus* (VISA) based on 14 gene parameters selected from 45 initial parameters [5]. Pesesky et al. compared rules-based algorithms to a machine-learning algorithm (logistic regression) for predicting AMR resistance profiles in *Enterobacteriaceae* [3]. The features used to build the prediction model were resistance genes determined by the AMR database Resfams [8]. Other studies used k-mers to represent bacterial genomes to build machine-learning models for AMR genotype identification and phenotype prediction [2, 6, 7].

**Fig. 1:**
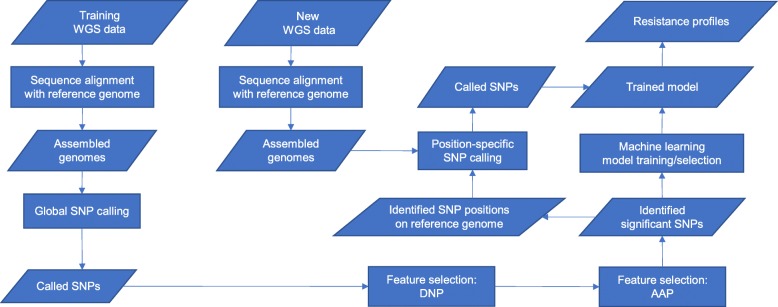
Workflow of the proposed machine learning approach to identify SNPs from WGS data. The prediction of AMR resistance profiles based on these identified SNPs is also part of the workflow. Although prediction is not the main purpose of this study, it is a natural next step after feature selection. In the figure, rectangles represent methodological steps, while parallelograms without right angles represent data or information. From the SNPs, resistance genes and other genetic elements can then be identified

In this paper, we propose an alternative to GWAS: use a completely data-driven feature selection method to identify significant SNPs. Compared to GWAS, this method needs no model assumptions and identifies SNPs based on their predictive powers. Then, these SNPs are used in two ways: (1) as markers to locate the genetic factors that affect AMR phenotypes, like SNPs from GWAS; (2) as features to build predictive models. Although the machine learning methods mentioned above start from WGS data, they only use WGS data to find known AMR-associated genes, and use these genes as predictors in their models. They ignore other novel genetic factors potentially associated with AMR. In comparison, in this study, the whole-genome SNP data (generated from WGS data) are directly used as input to our deep neural networks for feature selection. In this way, significant SNPs (and thus genetic factors) are identified by our method, rather than being selected from an AMR database, such as CARD [9] or ARDB [10]. It is worth mentioning that by directly using whole-genome SNP data as input, our method can also identify SNPs that fall in intergenic regions such as regulatory elements or promoters and that can be putatively associated with AMR. After significant SNPs are identified, predictive models are built based on these features. The whole workflow is shown in Fig. 1.

The proposed method, DNP-AAP (deep neural pursuit – average activation potential), involves two steps. DNP is a generic method using deep neural networks to perform feature selection and prediction, specifically designed for low-sample, high-dimension data, such as WGS data and plant genotype data [11]. One problem for DNP is that when it is fed different training data as in *k*-fold cross-validation, it outputs different sets of features. This makes it hard to select the final set of features, especially when the maximum number of features to be selected is large. This happens because DNP is dealing with high dimensional data and it uses dropout regularization in the feature selection process. Averaging multiple dropout results can reduce gradient variance in neural network training. This helps to improve the stability of feature selection results, but the instability is inherent in the model. To provide a more consistent way to select the final set of features generated by DNP, we add a calculation of average activation potential (AAP) for each selected feature, and use this AAP as the criterion to rank the feature importance. Activation potential has also been used to select features in action recognition from videos [12].

We applied DNP-AAP on a published *N. gonorrhoeae* WGS data with minimum inhibitory concentration (MIC) phenotypes for five commonly used antibiotics. Our experiment results show that DNP-AAP can effectively identify known AMR determinants in *N. gonorrhoeae*, and discover new potential AMR determinants. Subsequently, the identified SNPs were used to build logistic regression classifiers and the prediction AUCs (area under the curve) range from 0.949 to 0.994 for five subsets tested in our experiments.

The goal of this research is to design and test a data driven, deep learning method (DNP-AAP) that can predict SNPs associated with antimicrobial resistance, rather than to conduct a systematic comparison of feature selection methods. However, it is still worthwhile to place the results from DNP-AAP within the context of other feature selection methods. To this end we compared the results from DNP-AAP to those when using two other popular feature selection methods, LASSO [13] and AdaBoost [14].

## Results

We now present the results we obtained by applying DNP-AAP to the *N. gonorrhoeae* dataset. In the following analysis, we use the genes that have been reported to associate with *N. gonorrhoeae* AMR as a reference standard to evaluate the efficacy of our model in identifying known genes from WGS data. To test the significance of the identified SNPs in terms of power to predict a resistance profile, a very simple and efficient logistic regression classifier was trained for each antibiotic with the identified SNPs as features to classify *N. gonorrhoeae* strains.

One parameter that needs to be determined is how many features (SNPs) should be selected when performing feature selection. There is no universal solution to this problem. One strategy is to do a sensitivity analysis and see how adding or removing a feature affects the prediction accuracy. Another way is to choose this number based on the capacity of wet lab experiment facilities. If a lab can handle 50 (for example) genes in one experiment, then one can set the number of features to be 50. The results presented in this paper were obtained with a mix of these two criteria; that is, the criterion to select SNPs was that either a minimum number was reached or the prediction accuracy stopped increasing (< 0.05). The minimum number of SNPs to be selected was set to 10. This number was chosen given preliminary experimental results showing that the genes in the reference standard (Table S2 in Additional file 1) were not always at the top of the result list, but they were usually within the top 10. It is normal to not always see the genes in the reference standard at the top of the result list; because DNP is a greedy algorithm, the SNPs selected earlier are not always the globally optimal results.

The deep neural network (DNN) architecture was determined based on the structure suggested by Liu et al. [11] and preliminary investigations. According to Liu et al., the DNN that performs best in identifying known genes is a 4 layer neural network with 2 hidden layers. They also showed that changing the number of neurons in the hidden layers does not make much difference in identifying the known genes. Thus we used a simple DNN with the structure “ 41502/50^′′^−30−20−2, corresponding to the number of neurons in the input–hidden1–hidden2–output layers. The 2 neurons in the output layer correspond to our binary classes, susceptible and resistant to an antibiotic. 41502 in “ 41502/50” is the number of neurons used in the back propagation step, but not in the feed forward step. Every time a new feature is added to the selected set $\mathcal {S}$, the subnetwork, $|\mathcal {S}|-30-20-2$, is trained. In all our analyses, fifty features, including the bias item, were selected in each cross-validation for every antibiotic. Thus the final neural network that was trained had the structure 50−30−20−2.

### Ciprofloxacin resistance analysis

We first tested DNP-AAP on the ciprofloxacin resistance dataset which includes 302 susceptible and 364 resistant strains. Given the criterion to determine the number of SNPs to report, ten SNPs with the highest AAP were identified and are listed in Table 1. Gene annotations are from the reference genome NCCP11945 from EnsemblBacteria [15]. The annotation using NCBI is listed in Additional file 1: Table S3.

**Table 1:** SNPs identified for the resistance to ciprofloxacin (CIP) by DNP-AAP

ID Range	ID	AAP	Genes	Annotations	Known
[18797,18817]	18799	0.658	*gyrA*	DNA gyrase subunit A	✓
[4309,4366]	4363	0.536	*parC*	DNA topoisomerase IV subunit A	✓
	5087	0.506		intergenic between NGK_0295 and NGK_0296 ^∗^	
	5075	0.497	NGK_0295	glutathione synthetase	
	34282	0.483		intergenic between NGK_2199 and NGK_2200 ^∗^	
	33843	0.482	NGK_2182	putative integral membrane protein	
	20553	0.478	NGK_1395	OTB_PSEPK Probable sugar efflux transporter	
	2285	0.477	NGK_0116	conserved hypothetical protein	
	34301	0.475	NGK_2201	hypoxanthine-guanine phosphoribosyltransferase	
	16353	0.447	NGK_1090	conjugal transfer pilus assembly protein TraD	

Annotations are from EnsemblBacteria. The column “ID Range” lists the ranges of SNPs that fall in known AMR-associated genes (only) in our data. ID: ID of Identified SNP.

^*^NGK_0295: glutathione synthetase; NGK_0296: diacylglycerol kinase (DagK); NGK_2199: PtsH; NGK_2200: putative sugar transport PTS system IIA protein

Two genes associated with ciprofloxacin resistance, *gyrA* and *parC*, were identified by DNP-AAP, and the order of their importance also matches the published results [16]. The point mutation S91F (amino acid substitution) in *gyrA* was detected, while for *parC*, P88S was identified instead of the usually reported S87R, though both are present in resistant strains. The mutations in both gyrA and parC proteins can decrease the affinity between ciprofloxacin molecule and its binding sites, thereby conferring resistance to the antibiotic.

The SNP with ID 33843 falls in the gene NGK_1282, which encodes a putative integral membrane protein (GeneBank) in *N. gonorrhoeae*. KEGG Orthology (K07243) shows that this protein is a high-affinity iron transporter. Duncan [17] showed that ciprofloxacin kills bacteria by a mechanism involving production of hydroxyl radicals (·OH) from the Fenton reaction [18] and metabolic stress. The way for bacteria to avoid being killed is either by inhibiting the Fenton reaction through reducing ferrous iron (Fe ^2+^) or by reducing hydroxyl radicals (·OH) produced by the Fenton reaction after the addition of antibiotics. Although it is not clear how the pathway involving the gene NGK_1282 works, it seems possibly relevant to this antibiotic resistance mechanism. Two SNPs with ID 5087 and 34282 that fall in intergenic regions were also identified.

### Cefixime resistance analysis

The SNPs identified for cefixime resistance are shown in Table 2. The most significant mutations associated with cefixime resistance happen in the mosaic *penA* gene. Several *penA* SNPs were always selected with the highest AAP values. This shows that DNP-AAP can effectively identify these significant features contributing to cefixime resistance. DNP-AAP also identified several point mutations in two 16S RNA proteins which have been shown to be associated with azithromycin resistance [16, 19].

**Table 2:** SNPs identified for the resistance to cefixime (CFX) by DNP-AAP

ID Range	ID	AAP	Genes	Annotations	Known
	31799	0.423	NGK_rrna16s3	NGK_rrna16s3	
[28398,28481]	28431	0.419	*penA*	penicillin-binding protein 2	✓
[28398,28481]	28418	0.406	*penA*	penicillin-binding protein 2	✓
	29914	0.402	NGK_rrna16s2	NGK_rrna16s2	
[28398,28481]	28417	0.382	*penA*	penicillin-binding protein 2	✓
[28398,28481]	28428	0.382	*penA*	penicillin-binding protein 2	✓
	29915	0.376	NGK_rrna16s2	NGK_rrna16s2	
	29916	0.370	NGK_rrna16s2	NGK_rrna16s2	
[28398,28481]	28427	0.368	*penA*	penicillin-binding protein 2	✓
[28398,28481]	28429	0.367	*penA*	penicillin-binding protein 2	✓

Annotations are from EnsemblBacteria. The column “ID Range” lists the ranges of SNPs that fall in known AMR-associated genes (only) in our data. ID: ID of Identified SNP

### Penicillin resistance analysis

As for penicillin resistance, the gene *ponA*, which has been reported as being associated with penicillin resistance, was among the 10 locations of SNPs output by DNP-AAP (Table 3). Specifically, the SNP with ID 2755 leads to an amino acid substitution L421P in *ponA* product penicillin-binding protein 1A (PBP1). This mutation decreases penicillin acylation of PBP1 and increases penicillin resistance [16]. The SNP with the highest AAP value is in a conserved hypothetical protein, the function of which is not yet determined. The SNP with the second highest AAP falls in the gene NGK_2170 which encodes the outer membrane protein PIIc. GO (gene ontology) terms describe PIIc as “enables porin activity; involved in trans-membrane transport; part of membrane; part of integral component of membrane”. This is an interesting finding because one AMR mechanism is antibiotic efflux that can be conferred by membrane and membrane-associated proteins. These proteins can pump antimicrobial compounds out of microbial cells [20]. Another SNP, one with ID 10120, falls in a putative phage-associated gene NGK_0679. A bacteriaphage is a virus that infects and replicates within bacteria [21]. Bacteriaphages are one of the mobile genetic elements considered in the AMR studies of *N. gonorrhoeae* (see [22] and references therein). Bacteriaphages were also examined in other AMR studies [23, 24].

**Table 3:** SNPs identified for the resistance to penicillin (PEN) by DNP-AAP

ID Range	ID	AAP	Genes	Annotations	Known
	38424	0.344	NGK_2469	conserved hypothetical protein	
	33601	0.342	NGK_2170	outer membrane preprotein PIIc	
	18799	0.330	*gyrA*	DNA gyrase subunit A	
	29502	0.322	NGK_1906	monofunctional biosynthetic peptidoglycan	
				transglycosylase	
	29504	0.251	NGK_1906	monofunctional biosynthetic peptidoglycan	
				transglycosylase	
[2749,2763]	2755	0.236	*ponA*	penicillin-binding protein 1A	✓
	35095	0.219	NGK_2270	adhesin MafA	
	10120	0.213	NGK_0679	putative phage associated protein	
	40335	0.204		intergenic between NGK_2581 and NGK_2582 ^∗^	
	6817	0.203	NGK_0423	23S rRNA pseudo-uridine 1911/1915/1917	
				synthase	

Annotations are from EnsemblBacteria. The column “ID Range” lists the ranges of SNPs that fall in known AMR-associated genes (only) in our data. ID: ID of Identified SNP

^*^NGK_2581: Putative hemoglobin receptor component precursor HpuA; NGK_2582: Conserved hypothetical protein

Although effects of these mutations on penicillin resistance need further investigation, they seem relevant and can make promising candidates for microbiological experiments.

### Tetracycline resistance analysis

A SNP in the gene *rpsJ* associated with tetracycline resistance was identified by DNP-AAP (Table 4). The identified SNP (with ID 37927) leads to the amino acid substitution V57M in the encoded ribosomal protein S10, which reduces the affinity between tetracycline and the 30S ribosomal target [16]. The other observation regarding tetracycline resistance is that two genes encoding putative phage proteins are potentially implicated, each with two SNPs identified among the ten output from DNP-AAP. As mentioned before, bacteriaphages could potentially contribute to bacteria resistance (see references above). More verification is needed to see if these implicated genes contribute to tetracycline resistance.

**Table 4:** SNPs identified for the resistance to tetracycline (TET) by DNP-AAP

ID Range	ID	AAP	Genes	Annotations	Known
	27095	0.470		intergenic between NGK_1771 and NGK_1772 ^∗^	
	21468	0.205	NGK_1458	putative phage associated protein	
[37926,37927]	37927	0.196	*rpsJ*	30S ribosomal protein S10	✓
	29960	0.159	NGK_1968	IS1016 transposase	
	37300	0.150	NGK_2398	methionyl-tRNA formyltransferase	
	40041	0.131	NGK_2557	hemoglobin/transferrin/lactoferrin receptor	
				protein	
	21467	0.121	NGK_1458	putative phage associated protein	
	9785	0.120	NGK_0668	putative phage associated protein	
	9787	0.120	NGK_0668	putative phage associated protein	
	18761	0.119	NGK_1227	putative HTH-type transcriptional regulator	
				NMB1378	

Annotations are from EnsemblBacteria. The column “ID Range” lists the ranges of SNPs that fall in known AMR-associated genes (only) in our data. ID: ID of Identified SNP

^*^NGK_1771: transferrin-binding protein A; NGK_1772: TbpB

### Azithromycin resistance analysis

DNP-AAP did not identify any known genes associated with azithromycin resistance among the output SNPs given the selection criterion (Table 5). However, it identified a putative drug resistance gene NGK_1793 with the second highest AAP value. In addition, a SNP falling in the gene NGK_2342, which encodes pilC protein, is identified. pilC is the adhesion protein located at the tip of a bacterium pilus. Research shows that pilC can act on the bacterial cell surface and cooperate in DNA recognition and/or outer membrane trans-location [25]. Dötsch et al. [26] reported that mutations in pilC can increase drug resistance in *Pseudomonas aeruginosa*. Thus there is potential that this mutation can also relate to *N. gonorrhoeae* AMR.

**Table 5:** SNPs identified for the resistance to azithromycin (AZM) by DNP-AAP

ID Range	ID	AAP	Genes	Annotations	Known
	27421	0.424	NGK_1776	conserved hypothetical protein	
	27690	0.420	NGK_1793	putative drug resistance protein	
	30659	0.300	NGK_2022	Infection response protein Irg2	
	36328	0.294	NGK_2342	pilC protein	
	36810	0.290		intergenic between NGK_2354 and NGK_2355 ^∗^	
	30434	0.278		intergenic between NGK_1994 and NGK_1995 ^∗^	
	21513	0.269	NGK_1463	putative phage associated protein	
	39676	0.266	NGK_2537	homoserine kinase	
	36809	0.258		intergenic between NGK_2354 and NGK_2355 ^∗^	
	29095	0.254	NGK_1872	phosphatidylglycerophosphatase A	

Annotations are from EnsemblBacteria. The column “ID Range” lists the ranges of SNPs that fall in known AMR-associated genes (only) in our data. ID: ID of Identified SNP

^*^NGK_2354: Conserved hypothetical protein; NGK_2355: Hypothetical protein; NGK_1994: TspB2; NGK_1995: putative phage associated protein

### Prediction accuracy

ROC (receiver operating characteristic) curves and the average AUCs (Area Under the Curve) calculated from 5-fold cross-validation were used as a measure of the predictive power of the identified SNPs. A simple and efficient logistic regression classifier implemented using *scikit-learn* was trained with the identified SNPs. Although DNP performs classification simultaneously with feature selection, a separate classifier is built because the final identified features are selected with AAP from the aggregate candidate features from multiple experiments with cross-validation.

**Fig. 2:**
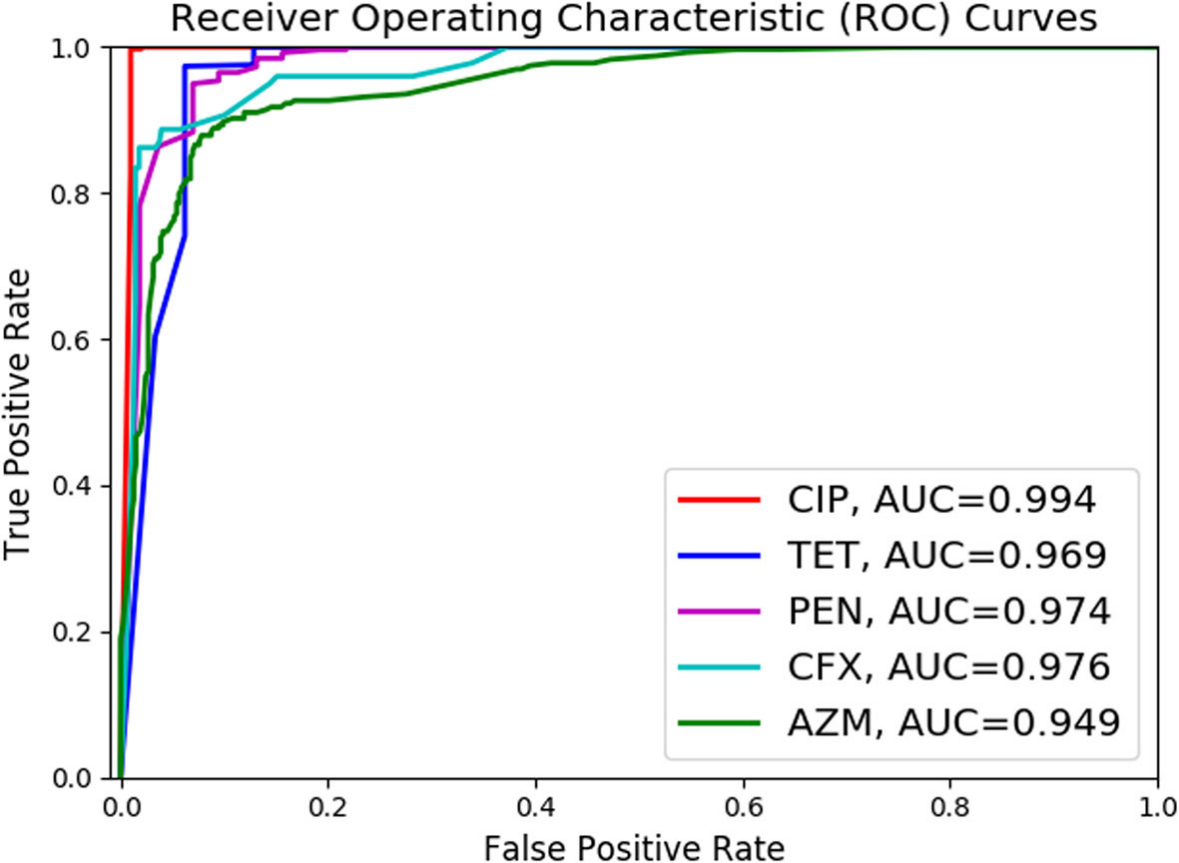
ROC curves and AUCs for the predicted resistance profiles for the five antibiotics under consideration

The ROC curves and AUCs generated by logistic regression with 5-fold cross-validation for the five antibiotic datasets are shown in Fig. 2. Of note is that the significant SNPs were identified with strains most resistant/susceptible to each antibiotic (statistics in Table 6), while the ROC curves and AUCs were obtained by considering the whole dataset with intermediate strains removed (statistics in Table 7).

**Table 6:** Counts of *N. gonorrhoeae* strains for each antibiotic

AMR/Antibiotics	CIP	AZM	TET	CFX	PEN
Susceptible	302	≤0.1	≤0.25	≤0.005	≤0.06
		45	26	75	46
Resistant	364	≥16	≥50	≥0.25	≥6
		38	26	108	37
Total number	666	83	52	183	83

*N. gonorrhoeae* strains for each antibiotic are balanced by selecting strains with the lowest and the highest MIC values. Criteria for selection are given above each count

**Table 7:** Summary of original antibiotic resistance data for *N. gonorrhoeae* strains

AMR/Antibiotic	CIP	AZM	TET	CFX	PEN
Susceptible	302	443	26	557	258
Intermediate	5	0	124	0	363
Resistant	364	233	526	108	46
Total number	671	676	676	665	667

There are 676 strains in total. MIC values were available for most strains for all five antibiotics. The numbers under each antibiotic are the counts in each category, obtained based on its CLSI breakpoints. CIP: ciprofloxacin; CFX: cefixime; PEN: penicillin; TET: tetracycline (TET); AZM: azithromycin (AZM)

Table 8 presents the true positive rate (TPR) for the classification of resistant strains given different false positive rates (FPR). TPR measures the proportion of resistant strains that are correctly classified as such and FPR measures the proportion of susceptible strains that are classified wrongly as resistant. When FPR is controlled around 10%, about 98%, 95%, 91% and 89% of resistant strains can be correctly classified for TET, PEN, CFX and AZM respectively. The reasons behind the differences in trends exhibited in Table 8 are not clear and deserve further investigation.

**Table 8:** TPR (=TP/(TP+FN)) for each antibiotic resistance prediction given different FPR (=FP/(FP+TN))

Drug/FPR	0.05	0.10	0.15	0.20
CIP	1.00	1.00	1.00	1.00
TET	0.74	0.98	1.00	1.00
PEN	0.86	0.95	0.98	0.996
CFX	0.89	0.91	0.96	0.96
AZM	0.76	0.89	0.92	0.93

CIP: Ciprofloxacin; AZM: azithromycin; TET: tetracycline; CFX: cefixime; PEN: penicillin

**Fig. 3:**
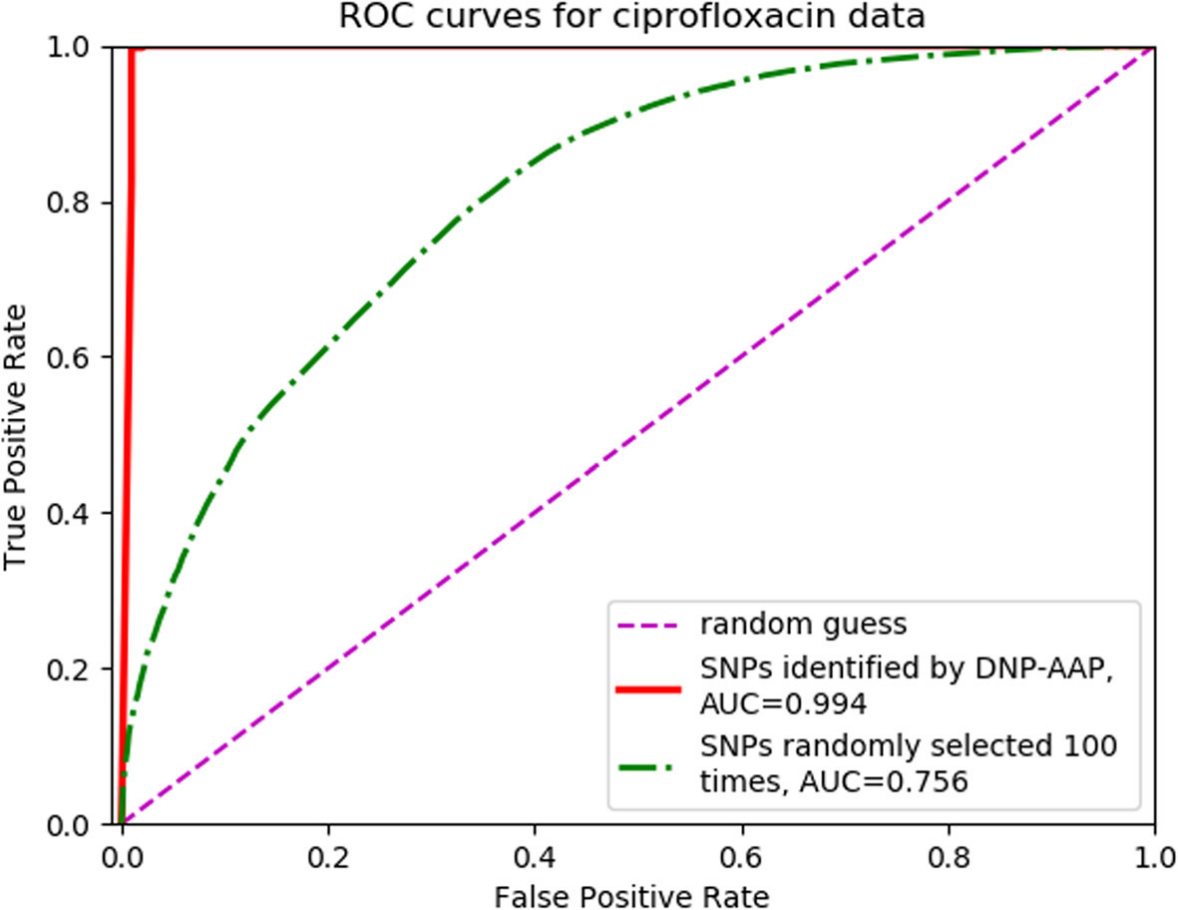
Classification performance of SNPs identified by DNP-AAP versus randomly selected SNPs. Shown are ROC curves for classifications made with SNPs identified by DNP-AAP and with randomly selected SNPs for ciprofloxacin data. The latter curve was obtained by randomly selecting 10 SNPs 100 times and averaging the resultant FPR (false positive rate) and TPR (true positive rate) values

To further show the predictive power of the identified SNPs, we compared the ROC curves and AUCs obtained by using the identified SNPs and the same number of SNPs randomly selected as features to build the logistic regression classifier. The ciprofloxacin dataset is used as an example here, and the results were similar for the other four antibiotics. It can be seen from Fig. 3 that SNPs identified by DNP-AAP were substantially better at predicting the AMR resistance of ciprofloxacin than the same number of randomly selected SNPs.

### Distribution of AAP

**Fig. 4:**
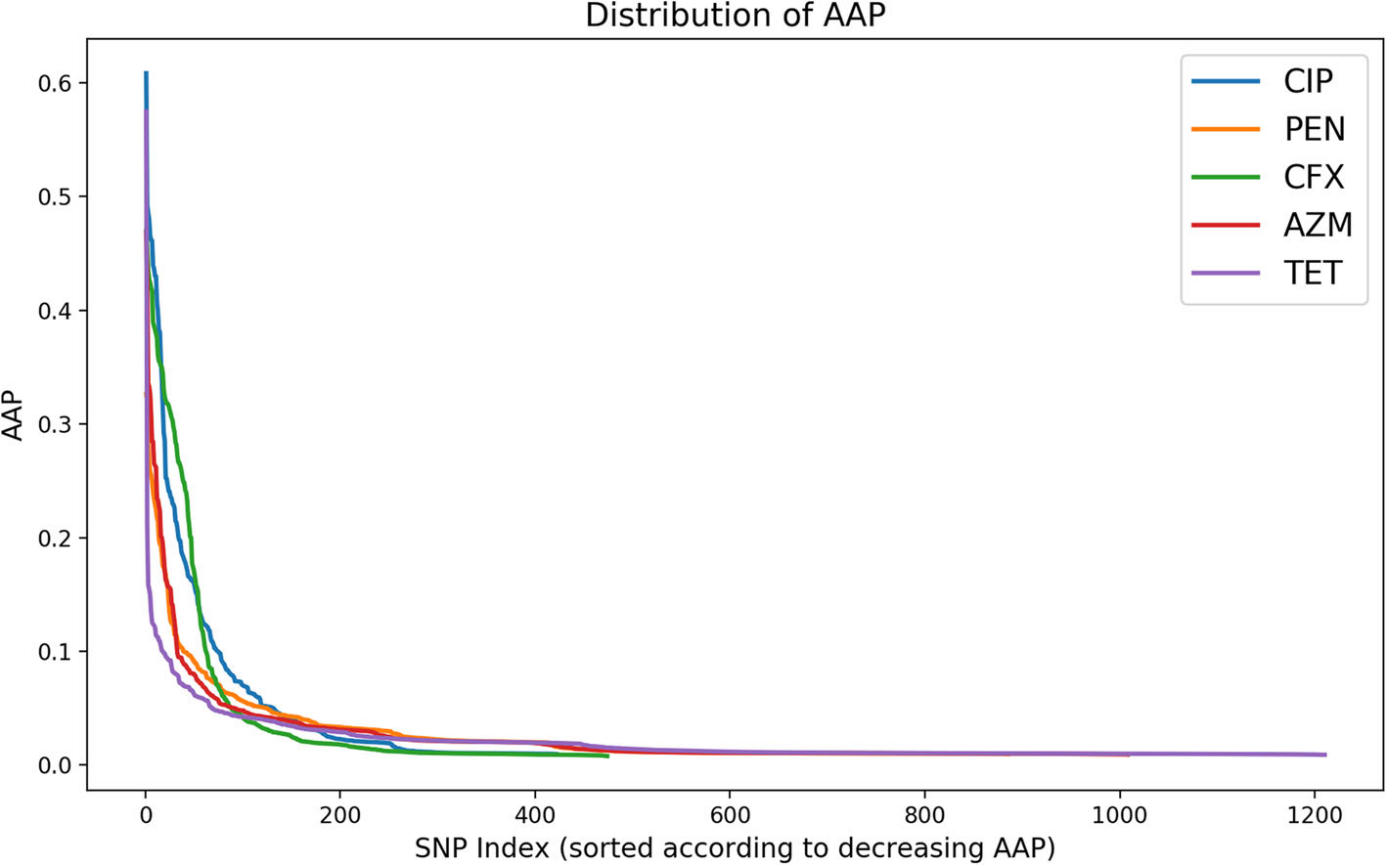
Distribution of average activation potentials (AAP) for the five antibiotic datasets

Average activation potentials (AAP) can be calculated between any layers in a deep neural network. We calculated AAPs between the input layer and the first hidden layer because direct correlation between the input features and their contribution to the whole neural network can only be established in this layer [12]. Figure 4 shows the input features sorted in the decreasing order of AAPs. Most of the selected input features from the 5-repeat experiments with 10-fold cross-validation had AAP close to zero, while only the first few inputs had significantly larger AAPs. These inputs contribute most to the activation of neurons in the neural network. The tails of the AAP distributions demonstrate the degree of selection consistency of the input features. On closer inspection, we can see that the total number of selected input features for ciprofloxacin is the smallest and the one for tetracycline is the largest. The shorter the tail, the more stable are the features output from DNP-AAP. However, since we are usually most interested in the top few (for example, 50 or 100) output SNPs, our DNP-AAP method provides good stability in identifying the most significant features.

### Performance of comparison techniques

As for DNP-AAP, the best SNPs from LASSO and AdaBoost and for each drug were examined to identify whether they were located within genes known to be associated with antimicrobial resistance. As shown by Additional file 1: Table S4, with one exception DNP-AAP reports the same number or more SNPs than the comparison methods.

Following the procedure for SNPs from DNP-AAP, logistic regression classifiers were constructed for each drug using the best SNPs identified by LASSO and AdaBoost, and the accuracies of the predictors were determined. The AUC values are given in Additional file 1: Table S5, while the ROC curves themselves are given in Additional file 1: Figures S3 and S4. As shown in the table, DNP-AAP performs better than LASSO and AdaBoost in a majority of cases.

## Discussion

It can be seen that the general predictive power of the identified SNPs is fairly strong for the five antibiotic resistance profiles. The SNPs for ciprofloxacin show the strongest predictive power, yielding the AUC of 0.994, while SNPs identified for azithromycin resistance show the weakest predictive power, yielding the AUC of 0.949. One possible explanation for the difference in predictive power among the drugs is the amount of data available for each in the various resistance categories. For example, as shown in Table 7, the data for ciprofloxacin – the drug with the best predictive power – was well-balanced and numerous in both the susceptible and resistant categories. However, the data for the other drugs was less balanced. Filtering to obtain a better balance between the susceptible and resistant categories (see Table 6) resulted in less data for training. The reduction in data quantity might be the cause of the reduced predictive power.

The purpose of tools such as DNP-AAP is to provide microbiologists with a list of candidate genes and other genetic factors. They can further distill these candidates by applying their domain knowledge with the aim of improving their experimental productivity. Although the new potential determinants are strongly predictive of AMR resistance in *N. gonorrhoeae*, their functions need to be verified by further examination.

## Conclusions

In biology, phenotypes are determined by genotype and the interaction between genotype and environment. Thus, by looking into genomic variations between individuals, we can identify contributors to their phenotypic differences. This is why SNPs are commonly used as markers to study the genetic cause of diseases and antimicrobial resistance, and also used in plant and animal breeding programs to select superior varieties. SNPs can be tracked and quantified over time, so they are also used to study evolutionary change in populations.

In this work, we propose DNP-AAP to identify known and discover new potential AMR-associated point mutations from whole-genome SNP data. This step can serve as a starting point of building machine learning models for AMR resistance profile prediction based on whole-genome genotype data. We also propose a general workflow to build machine learning models for AMR prediction from WGS data (shown in Fig. 1). The advantages of this workflow include: (1) it is generic and completely data-driven; (2) AMR predictors identified are not limited to known genes from AMR databases and new, putative AMR-associated genes and intergenic regions can be identified; (3) once significant predictors are identified, only position-specific SNP calling needs to be performed for the AMR resistance prediction of new samples; (4) it is easy to monitor the development of point mutations when new WGS and resistance phenotype data becomes available.

In order to test the efficacy of DNP-AAP, we applied it to a *N. gonorrhoeae* WGS data with resistance profiles to five commonly used antibiotics for gonorrhoea treatments. The results show that DNP-AAP can effectively identify known AMR-associated SNPs for the antibiotics ciprofloxacin, cefixime, penicillin and tetracycline. It also provides a list of candidate genes and intergenic regions that might lead to novel AMR factor discovery, though further verification is required. DNP-AAP can be applied to AMR analysis of any bacterial species with genomic variants and phenotype data. This can provide microbiologists with a useful screening tool to generate genetic candidates for further lab experiments.

Despite the promising results, DNP-AAP has limitations. First, it cannot provide explicit evidence of the interactions between SNPs, although the neural network accounts for non-linear relationships between neurons. Second, the features identified can vary depending on the training data and also the number to be output from DNP. This stochastic characteristic of the algorithm should not deter others from applying this method, however, since the features identified with high rank by AAP are relatively stable.

## Methods

DNP-AAP is a quantifying method to select features from low-sample, high-dimension data. In this section, we will introduce DNP and AAP in more detail.


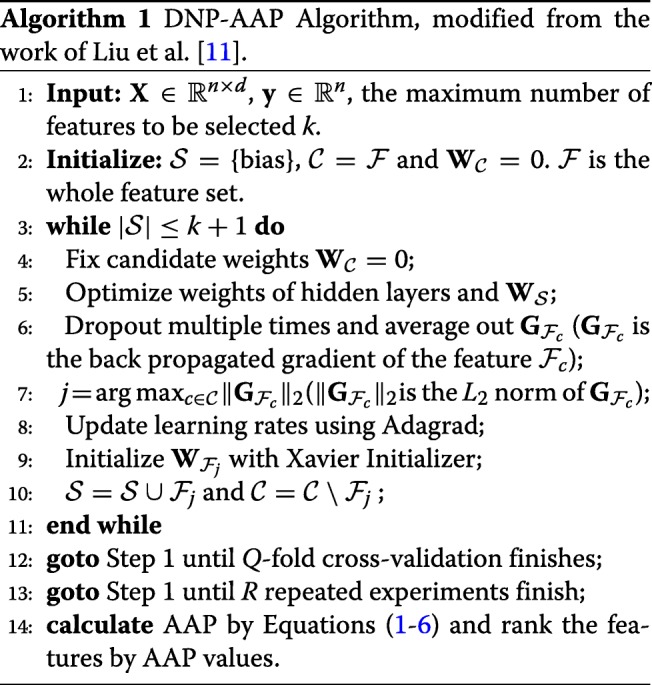


### Preliminary feature selection with DNP

DNP provides a general way to select features from high-dimension, low-sample size data within a deep neural network (DNN) architecture, which makes it possible to apply DNNs to problems such as plant phenotype prediction from genotype and antimicrobial resistance prediction based on WGS data. Both problems suffer from insufficiency of samples while being burdened with high-dimension data. Essentially, DNP applies conventional forward feature selection to deep neural networks using back propagated gradients. It starts with initializing a selected set $\mathcal {S}$ with a bias node added to the input layer so that not all neurons in the DNN are inactive; that is, $\mathcal {S}=\{bias\}$. This means that only weights connected to the bias node are initialized with values, while the weights for all other features are set to 0. Then features in the candidate set $\mathcal {C}$ are selected one by one according to the *L*_2_ norm of their back-propagated gradients. The higher the norm, the more the change of the candidate feature’s weights contributes to minimizing the cost function in neural network training, and thus the feature is removed from $\mathcal {C}$ and added to $\mathcal {S}$. This process is illustrated in Additional file 1: Figure S1.

The way DNP selects features is similar to the grafting algorithm proposed by Perkins et al. [27] where, in each iteration, the feature with the largest norm of back propagated gradient is added from a candidate set to a selected set. Both DNP and the grafting algorithm are greedy because they can only ensure the feature selected is the best at this point but cannot guarantee that the final set of features is the global optimum set. A simple description on why back-propagated gradients can be used to select features is given in Section S1 of Additional file 1.

DNP adopts dropout on hidden layers to reduce the high variance of back propagated gradients when dealing with small-sample data. Although dropout can also be applied on the input layer, in practice, this is usually not performed because it will directly discard information from input data. Especially in feature selection settings, we want to keep all the features in the input layer so that we do not lose any important features during random dropouts. In each iteration to select one feature, dropouts are performed multiple times, and each candidate feature’s back propagated gradient is averaged over all dropouts. This can help to reduce gradient variance and add some stability to feature selection. The DNP process is illustrated in the first 11 lines in Algorithm 1.

### Feature importance ranking with AAP

**Fig. 5:**
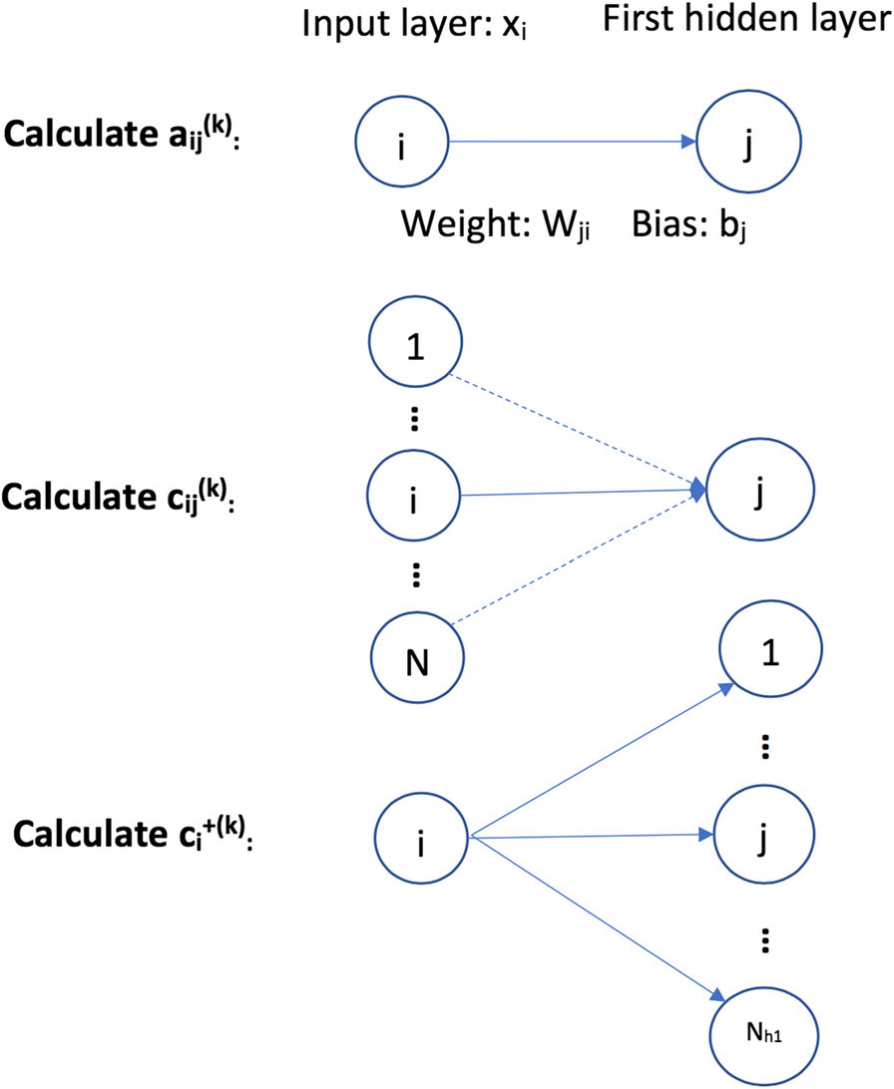
The main steps in defining average activation potential (AAP)

In order to evaluate the contribution of each identified feature to a prediction model, a quantitative metric is required to rank the importances. To this end, we use a concept called average activation potential (AAP) [12] as the metric to evaluate the importance of a feature selected by DNP. For each input feature, AAP calculates its activation potential on each neuron in the first hidden layer, and averages this potential among all training samples. Then, the total activation potential of this input variable is the sum of its activation potential on all the neurons in the first hidden layer. Since DNP is a stochastic algorithm, to further improve the consistency of identified features, we run multiple repeated experiments on each dataset. Therefore, AAP is also averaged on multiple experiment results. Intuitively, the more a feature is selected by cross-validation in multiple experiments, the more likely it is significant. The definition of AAP is given next and its main steps are shown in Fig. 5.

To define the activation contribution of a node *i* in the input layer to all nodes in the first hidden layer, $c_{i}^{+}$, we first define its activation potential to one node *j* in the first hidden layer given one training instance *k*, $a_{ij}^{(k)}$,

1 $$ a_{ij}^{(k)} = w_{ji}^{k} * x_{i}^{k} + b_{j}^{k}   $$

where $x_{i}^{k}$ is the *i*^*th*^ input feature value of the *k*^*th*^ training instance, and $w_{ji}^{k}$ is the weight between node *i* and *j*, and $b_{j}^{k}$ is the bias item to node *j* given instance *k*. This is the first step “Calculate $a_{ij}^{(k)}$” in Fig. 5. Now we define the average absolute activation potential of node *i* to node *j* given all the training instances:

2 $$ {aa}_{ij} = \frac{1}{M} \sum_{k=1}^{M} |{a_{ij}^{(k)}}|  $$

where *M* is the number of training instances. The absolute value is used to penalize nodes with large negative depression of the nodes in the next layer. Now we use *aa*_*ij*_ to define $c_{ij}^{k}$, the contribution of node *i* to the activation of node *j* given training instance *k*, which is shown as follows:

3 $$ c_{ij}^{(k)} = \frac{a_{ij}^{(k)}}{\sum_{p=1}^{N} {aa}_{pj}}  $$

where *N* is the number of nodes in the input layer. Before we define the final activation contribution of node *i* in the input layer to all nodes in the first hidden layer, we briefly introduce the activation function used in neural network training. An activation function performs non-linear transformation to input features. This makes a neural network capable of learning and solving more complex tasks. A neural network without an activation function is just a linear regression model. One commonly used activation function in DNN training is a Rectifier Linear Unit (ReLU), which is also used in this work. A node is activated when its output value is greater than 0; otherwise, it is not activated. The following is the ReLU function:

$$ReLU(x) = max(0, x) $$

Given the ReLU activation function, we define the positive activation contribution of node *i* to the whole neural network given the *k*^*th*^ training instance as follows:

4 $$ c_{i}^{+(k)} = \sum_{j=1}^{N_{h1}} ReLU\left(c_{ij}^{(k)}\right)  $$

This is reasonable because when ReLU is used as the activation function, any nodes in a hidden layer with negative output values are set to be inactive, and these nodes will not contribute to the final training of the neural network. Thus, we only count the positive contribution of input features. Then the activation potential of node *i* to the whole neural network given all training data is given as

5 $$ c_{i}^{+} = \frac{1}{M} \sum_{k=1}^{M} c_{i}^{+(k)}  $$

Due to the stochastic nature of DNP, to further increase the stability of DNP results, we rank the features based on multiple repeated experiments. Because of the small number of training instances, cross-validation is used in DNP training. Therefore, we average the activation potential of node *i* to the whole neural network among *R* repeated experiments and *Q*-fold cross validation,

6 $$ {AAP}_{i} = \frac{1}{R*Q} \sum c_{i}^{+(r, q)}   $$

and use this as the final criterion to rank feature importance. The superscript (*r*,*q*) refers to the *q*^*th*^ cross validation in the *r*^*th*^ experiment. The whole learning process of DNP-AAP is shown in Algorithm 1.

### Implementation

The DNP-AAP algorithm is implemented in Python Version 3 utilizing the deep learning package *MXNet*.

### Comparison

We compare the feature selection performance of DNP-AAP with two well-established methods for feature selection, LASSO [13] and AdaBoost [14]. Adaboost has been utilized for feature selection for antimicrobial resistance prediction using k-mers [2]. Here we apply this approach to SNP prediction in antimicrobial resistance genetic factor identification. blackImplementations of LASSO and AdaBoost are provided through the *linear_model.lasso* and *AdaBoostClassifier* packages from *scikit-learn* version 0.20.2, respectively. *lasso* is run with default parameters except for *alpha*, which is set to 0.01 to achieve a number of reported SNPs comparable to that from DNP-AAP. For *AdaBoostClassifier* the following parameters are specified: decision tree classifiers of maximum depth of 1 are used as weak learners; the maximum number of weak learners is set to be 100; 1 is used as the learning rate; and the learning algorithm is set to “SAMME.R”.

### Whole-genome sequence data

Whole-genome sequence data of *N. gonorrhoeae* with antimicrobial susceptibilities to five commonly used antibiotics from three published studies [19, 28, 29] were downloaded from NCBI Sequence Read Archive (SRA) [30]. The NCBI identifiers of all strains are listed in Additional file 2. The steps to preprocess the WGS data are outlined as follows:

- Paired-end short reads were downloaded from NCBI SRA [30] with *fastq-dump*.
- Sequence alignments were performed with *BWA MEM* [31] using NCCP11945 [32] as reference genome.
- *sam* files generated from *BWA MEM* were transformed to *bam* format with *SAMtools* [33, 34].
- Variant calling was performed using *Freebayes* [35] with parameters set as in other studies which also used *Freebayes* for SNP calling in *N. gonorrhoeae* [19].
- Variant calling results were filtered with *Freebayes* setting ‘vcffilter -f ~TYPE = snp~’ to retain only SNP data.

Eventually, we generated a dataset with 676 samples, each of which had 41502 SNPs. A SNP is a variation at a single position on the DNA sequences of different individuals. A variation is considered as a polymorphism only when it is detected above a certain threshold such as 1% or 5% in a population. Such a constraint [19] is used here to exclude variations arising from errors or very rare mutations.

SNPs usually take values 0 (the same as reference allele), 1 (the alternative allele), and “.” (missing data). However, *Freebayes* also generates numbers larger than 1 for some positions. This means that it finds multiple alleles at those positions. We replaced the numbers > 1 with 1 to only show that there is variation at those positions. As for missing values, we did not try to impute them, but rather assigned them the value 0.5 (simply the mean of 0 and 1) instead of following the example in GAPIT [36], which replaces missing values with 0 or 1 by simple imputations. The reason for not imputing missing values is that our sample is not big enough to make a verifiable imputation. Further, simple imputation methods, such as mode imputation, which fills the missing data with the most common value each SNP takes, can introduce bias into data favoring the strains with major SNPs.

### Antimicrobial resistance phenotype

Minimum inhibitory concentration (MIC) was used as a numerical measurement of AMR phenotype. It is the lowest concentration of a drug that will inhibit the visible growth of a microorganism [37]. In this study, two classes of *N. gonorrhoeae* strains were used, i.e., *susceptible* versus *resistant*, which were grouped based on their MIC values and the breakpoints (thresholds) given by Clinical Laboratory Standard Institute (CLSI) [38]. The MIC thresholds for the five antibiotics examined in the data are shown in Additional file 1: Table S1. The MIC distribution for each of the five drugs is given in Figure S2 of Additional file 1.

### Dataset for each antibiotic

As mentioned above, *N. gonorrhoeae* strains were grouped into *Susceptible* (S) or *Resistant* (R) classes based on their MIC values and CLSI breakpoints [38]. Based on the CLSI breakpoints for each antibiotic, we obtained five datasets, shown in Table 7. To simplify the description, *Decreased Susceptibility* for cefixime is also referred to as *Resistant* in this paper. The complete labeled data, including “Intermediate" (I) class, is summarized in Table 7.

From the clinical application perspective, we only considered the strains in S and R classes. It can be seen from the table that most sub-datasets were imbalanced except for ciprofloxacin. To increase the quality of the limited data for feature selection, we balanced the datasets by taking strains with the most extreme MIC values; i.e., susceptible strains were selected with the lowest MIC values, and resistant strains were selected with the highest MIC values. The thresholds used were the ones that yield approximately the same numbers of resistant and susceptible strains. The data statistics are summarized in Table 6. Ciprofloxacin data was approximately balanced and is listed in the table for completeness.

### Antimicrobial loci in *N. gonorrhoeae*

The genetic factors that have been reported to be associated with AMR in *N. gonorrhoeae* to the five antibiotics are summarized in Additional file 1: Table S2. There were no SNPs from plasmids in the data because only chromosomal DNAs were extracted for sequencing [19, 28, 29]. The plasmid genes are listed in the table for reference purposes.

## Additional files

Additional file 1 Supporting information. This PDF file includes: (1) **Section S1**: a description of how back propagated gradients in neural network training are used for feature selection; (2) **Figure S1** gives an illustration on how DNP works; (3) **Figure S2** shows the MIC distribution of the five drugs; (4) **Figure S3** provides the predicted resistance profiles and AUC values for the classifier built using SNPs identified by AdaBoost; (5) **Figure S4** shows the predicted resistance profiles and AUC values for the classifier built using SNPs identified by LASSO; (6) **Table S1** provides the CLSI breakpoints; (7) **Table S2** lists the known AMR loci of the five drugs examined in this study; (8) **Table S3** lists the SNPs output from DNP-AAP with NCBI annotations for the reference genome NCCP11945; (9) **Table S4** lists the numbers of SNPs in known chromosomal AMR determinants in Table 2 reported by DNP-AAP, AdaBoost, and LASSO; (10) **Table S5** lists the AUC values for the logistic regression classifiers built using the SNPs reported by each of DNP-AAP, AdaBoost, and LASSO. (PDF 394 kb)

Additional file 2 NCBI identifiers. This is a text (.txt) file with NCBI identifiers of the raw reads of 676 *N. gonorrhoeae* strains used and analyzed in this study. URLs at NCBI corresponding to those identifiers are also given. (TXT 45 kb)

## Abbreviations

AMR: Antimicrobial resistance
AUC: Area under the curve
AZM: Azithromycin
CFX: Cefixime
CIP: Ciprofloxacin
CLSI: Clinical laboratory standard institute
DNN: Deep neural network
DNP-AAP: Deep neural pursuit – average activation potential
FPR: False positive rate
GWAS: Genome-wide association study
MIC: Minimum inhibitory concentration
PEN: Penicillin
ROC: Receiver operating characteristic
SNP: Single nucleotide polymorphism
TET: Tetracycline
TPR: True positive rate
WGS: Whole-genome sequencing
