# Supplementary material for: Antimicrobial resistance genetic factor identification from whole-genome sequence data using deep feature selection
Source: BMC Bioinformatics. 2019 Dec 24;20(Suppl 15):535. doi: 10.1186/s12859-019-3054-4 (PMC6929425; doi:10.1186/s12859-019-3054-4)
Supplement: Supplementary file 1 — Supporting information. This PDF file includes: (1) Section S1: a description of how back propagated gradients in neural network training are used for feature selection; (2) Figure S1 gives an illustration on how DNP works; (3) Figure S2 shows the MIC distribution of the five drugs; (4) Figure S3 provides the predicted resistance profiles and AUC values for the classifier built using SNPs identified by AdaBoost; (5) Figure S4 shows the predicted resistance profiles and AUC values for the classifier built using SNPs identified by LASSO; (6) Table S1 provides the CLSI breakpoints; (7) Table S2 lists the known AMR loci of the five drugs examined in this study; (8) Table S3 lists the SNPs output from DNP-AAP with NCBI annotations for the reference genome NCCP11945; (9) Table S4 lists the numbers of SNPs in known chromosomal AMR determinants in Table 2 reported by DNP-AAP, AdaBoost, and LASSO; (10) Table S5 lists the AUC values for the logistic regression classifiers built using the SNPs reported by each of DNP-AAP, AdaBoost, and LASSO. (PDF 394 kb) [file 12859_2019_3054_MOESM1_ESM.pdf]

Supplementary Material

Antimicrobial resistance genetic factor identification from whole-genome sequence data using deep feature selection

Jinhong Shi[1], Yan Yan[1], Matthew G. Links[1,2], Longhai Li[3], Jo-Anne R. Dillon[4,5], Michael Horsch[1] and Anthony Kusalik[1*]

*Correspondence: tony.kusalik@usask.ca
[1]Department of Computer Science, University of Saskatchewan, 110 Science Place, S7N 5C9 Saskatoon, Canada
Full list of author information is available at the end of the article

Section 1: Back-propagated Gradients in Neural Network Training for Feature Selection

It is an optimization problem to find the weights of a neural network by minimizing its cost function. A general form of cost functions can be written as a summation of loss function and regularized items [1]:

$$J(w) = C(w) + \lambda_2 \sum_{i=1}^{p} \alpha_{2,i}|w_i|^2 + \lambda_1 \sum_{i=1}^{p} \alpha_{1,i}|w_i| \tag{1}$$

where

$$C(w) = \frac{1}{m}\sum_{i=1}^{m}(y_i - \sum_{j=1}^{p} x_{ij}w_j)$$

is the loss function that measures the difference between true values and predictive values. The second item in Equation (1) is a generalized form of L_2 regularization and the third one is L_1 regularization. The parameters λ_1 and λ_2 control the strength of penalization on the magnitude of coefficients in the trained model. A larger λ leads to a less complex model, and thus L_1 and L_2 regularization is an effective way to prevent overfitting in large complex model training. Usually, $\lambda_1, \lambda_2 \in [0, 1]$ and $\alpha_{1,i}, \alpha_{2,i} \in [0, 1]$.

Gradient descent is a technique used to find the solution to the optimization problem in Equation (1). Taking the derivative of it, and we get

$$\begin{aligned}\frac{\partial J(w)}{\partial w_i} &= \frac{\partial C(w)}{\partial w_i} + 2\lambda_2\alpha_{2,i}w_i + \lambda_1\alpha_{1,i}sign(w_i)\\ &= \frac{\partial C(w)}{\partial w_i} + 2\lambda_2\alpha_{2,i}w_i \pm \lambda_1\alpha_{1,i}\end{aligned}$$

For feature selection, the input features are divided into a candidate set $\mathcal{C}$, in which the weights of each feature are fixed to zero, and a selected set $\mathcal{S}$, in which the weights of each feature are optimized during the training of the subnetwork with

selected features in the input layer. Therefore, for each feature F_i in candidate set $\mathcal{C}$, its weight w_i is 0. Then we get

$$\frac{\partial J(w)}{\partial w_i} = \frac{\partial C(w)}{\partial w_i} \pm \lambda_1 \alpha_{1,i} \tag{2}$$

Notice that we replaced $sign(w_i)$ by ± 1. Derivative is not defined for the absolute value function $|x|$ at $x = 0$. However, here we mainly focus on which feature is to be selected so that Equation (1) is decreased the most when we adjust its weights away from 0 (adding it to $\mathcal{S}$ from $\mathcal{C}$).

When $\lambda_1 \alpha_{1,i} \geq 0$, if

$$\frac{\partial C(w)}{\partial w_i} > \lambda_1 \alpha_{1,i},$$

then

$$\frac{\partial J(w)}{\partial w_i} = \frac{\partial C(w)}{\partial w_i} + \lambda_1 \alpha_{1,i} > 0,$$

and

$$\frac{\partial J(w)}{\partial w_i} = \frac{\partial C(w)}{\partial w_i} - \lambda_1 \alpha_{1,i} > 0.$$

Therefore, to decrease $J(w)$, we need to decrease w_i, so we will get $w_i < 0$. Similarly, if

$$\frac{\partial C(w)}{\partial w_i} < -\lambda_1 \alpha_{1,i},$$

then

$$\frac{\partial J(w)}{\partial w_i} = \frac{\partial C(w)}{\partial w_i} \pm \lambda_1 \alpha_{1,i} < 0.$$

In this case, to decrease $J(w)$, we need to increase w_i, so we will get $w_i > 0$. In summary, when

$$|\frac{\partial C(w)}{\partial w_i}| > \lambda_1 \alpha_{1,i},$$

we can decrease $J(w)$ by adjusting w_i away from zero, while if

$$|\frac{\partial C(w)}{\partial w_i}| < \lambda_1 \alpha_{1,i},$$

we can only increase $J(w)$ no matter how we adjust w_i away from zero. This is why when the regularization parameter λ_1 is large, L_1 regularization will result in a

sparse model with many zero-valued weights. When only L_2 regularization is used, then Equation (2) becomes

$$\frac{\partial J(w)}{\partial w_i} = \frac{\partial C(w)}{\partial w_i} \tag{3}$$

In both Equation (2) and Equation (3), we can see that the larger the magnitude of $|\partial J(w)/\partial w_i|$, the more it will contribute to minimizing $J(w)$ by updating w_i from zero. This is why the norm of the back-propagated gradient for each feature in the candidate set can be used as the criterion for feature selection. DNP and grafting both used gradients in their feature selection algorithms [1, 2].

Figure 1: Illustration of how features are selected in DNP (deep neural pursuit).

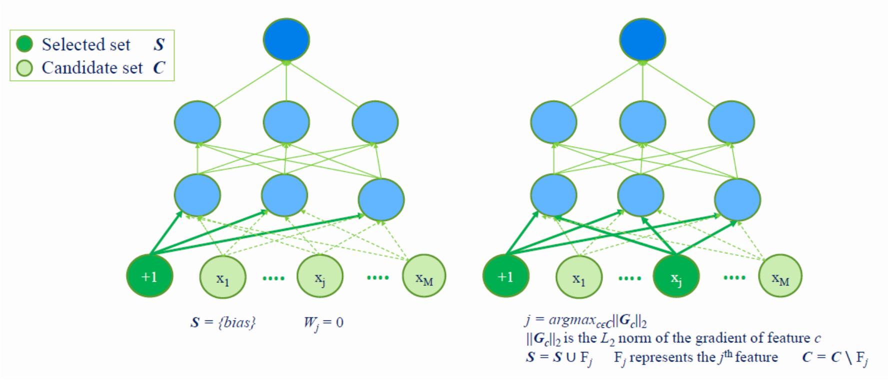

Figure 1 Features in candidate set $\mathcal{C}$ are selected one by one based on the L_2 norm of their back propagated gradients. Once selected, the feature is removed from the candidate set $\mathcal{C}$ and added to the selected set $\mathcal{S}$.

Figure 2: Histogram of MIC Distribution of the Five Antibiotics

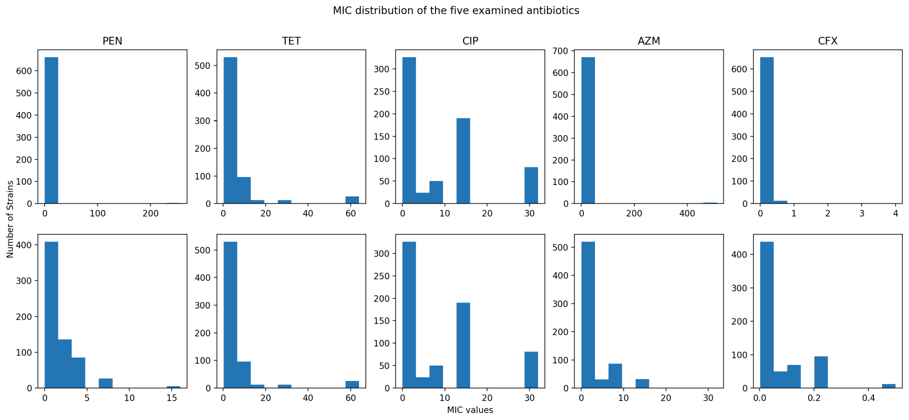

Figure 2 Histograms of the MIC distribution for each antibiotic. The top row shows the distribution of all MIC values for each antibiotic. The bottom row shows the distribution for PEN, AZM and CFX with extremely high MIC values removed. The MIC distributions of TET and CIP are added to the bottom row to fill it out. PEN, penicillin; TET, tetracycline; CIP, Ciprofloxacin; AZM, azithromycin; CFX, cefixime.

Figure 3: ROC curves and AUCs for the predicted resistance profiles for the five antibiotics under consideration using SNPs identified by AdaBoost.

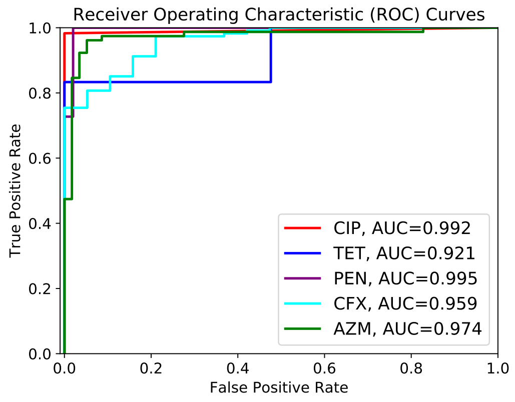

Figure 3 ROC curve and AUC for each antibiotic. CIP, Ciprofloxacin; TET, tetracycline; PEN, penicillin; CFX, cefixime; AZM, azithromycin.

Figure 4: ROC curves and AUCs for the predicted resistance profiles for the five antibiotics under consideration using SNPs identified by LASSO.

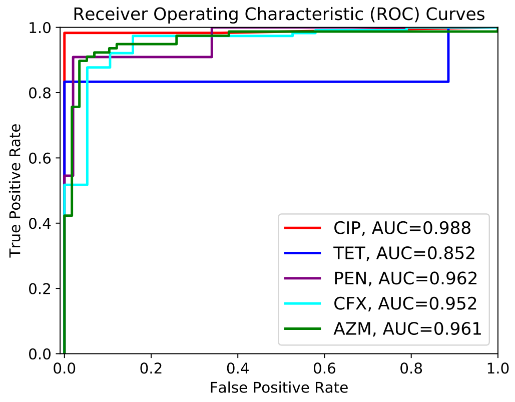

Figure 4 ROC curve and AUC for each antibiotic. CIP, Ciprofloxacin; TET, tetracycline; PEN, penicillin; CFX, cefixime; AZM, azithromycin.

Table 1 CLSI breakpoints for each antibiotic [3]

Antibiotic	MIC Interpretative Standards (ug/ml)			
	S	I	R	DS
PEN	≤ 0.06	$0.12 \sim 1.0$	≥ 2.0	
TET	≤ 0.25	$0.5 \sim 1.0$	≥ 2.0	
CIP	≤ 0.06	$0.12 \sim 0.5$	≥ 1.0	
AZM*	≤ 1.0		≥ 2.0	
CFX*	≤ 0.125			≥ 0.25

PEN, penicillin; TET, tetracycline; CIP, Ciprofloxacin; AZM, azithromycin; CFX, cefixime.
S = Susceptible, I = Intermediate, R = Resistant, DS = Decreased Susceptibility
*The breakpoints for AZM and CFX are from Centers for Disease Control and Prevention, 2007 [4] and World Health Organization 2012 [5], respectively.

Table 2 Chromosomal loci associated with antimicrobial resistance to the five antibiotics in *N. gonorrhoeae* examined in this work [6–8]. Plasmid genes are also listed, but only for reference purposes.

AMR elements \ Antibiotic	CIP	AZM	TET	CFX	PEN	Mechanisms
gyrA	✓					Antibiotic target alteration
parC	✓					
rpsJ			✓			
penA				✓	✓	
ponA				✓	✓	
23S rRNA		✓				
norM	✓					Antibiotic efflux
norM promoter	✓					
mtrR		✓	✓	✓	✓	
mtrR promoter		✓	✓	✓	✓	
macAB		✓				
penB (porB)			✓	✓	✓	Decrease in permeation across the outer membrane
penC (pilQ)				✓	✓	
erm(B, C, F) (plasmid)		✓				Plasmid mediated resistances
ere(A, B) (plasmid)		✓				
mef (plasmid)		✓				
bla_{TEM} (plasmid)					✓	
tetM (plasmid)			✓			

Table 3 SNPs identified for resistance to CIP, CFX, PEN, TET, and AZM by DNP-AAP. Annotations are from NCBI.

ID Range	ID	AAP	Genes	Annotations	Reported
				**Ciprofloxacin (CIP)**	
[18797, 18817]	18799	0.658	*gyrA*	DNA gyrase subunit A	✓
[4309, 4366]	4363	0.536	*parC*	DNA topoisomerase IV subunit A	✓
	5087	0.506		*intergenic between NGK_RS01270 and NGK_RS01275	
	5075	0.497	NGK_RS01270	glutathione synthetase	
	34282	0.483		*intergenic between NGK_RS09910 and NGK_RS09915	
	33843	0.482		*intergenic between NGK_RS09815 and NGK_RS09830	
	20553	0.478	NGK_RS00430	sugar transporter	
	2285	0.477	NGK_RS00430	RNA-binding protein	
	34301	0.475	NGK_RS09920	hypoxanthine-guanine phosphoribosyltransferase	
	16353	0.447	NGK_RS04915	conjugative coupling factor TraD, PFGI-1 class	
				**Cefixime (CFX)**	
	31799	0.423		*intergenic between NGK_RS09405 and NGK_RS09440	
[28398, 28481]	28431	0.419	*penA*	penicillin-binding protein 2	✓
[28398, 28481]	28418	0.406	*penA*	penicillin-binding protein 2	✓
	29914	0.402		*intergenic between NGK_RS08825 and NGK_RS13555	
[28398, 28481]	28417	0.382	*penA*	penicillin-binding protein 2	✓
[28398, 28481]	28428	0.382	*penA*	penicillin-binding protein 2	✓
	29915	0.376		*intergenic between NGK_RS08825 and NGK_RS13555	
	29916	0.370		*intergenic between NGK_RS08825 and NGK_RS13555	
[28398, 28481]	28427	0.368	*penA*	penicillin-binding protein 2	✓
[28398, 28481]	28429	0.367	*penA*	penicillin-binding protein 2	✓
				**Penicillin (PEN)**	
	38424	0.344	NGK_RS11280	CRISPR-associated protein Cas4	
	33601	0.342	NGK_RS09760	Opacity protein opA54	
	18799	0.330	*gyrA*	DNA gyrase subunit A	
	29502	0.322	NGK_RS08530	monofunctional biosynthetic peptidoglycan transglycosylase	
	29504	0.251	NGK_RS08530	monofunctional biosynthetic peptidoglycan transglycosylase	
[2749, 2763]	2755	0.236	*ponA*	penicillin-binding protein 1A	✓
	35095	0.219	NGK_RS10250	adhesin MafA	
	10120	0.213	NGK_RS03045	hypothetical protein	
	40335	0.204		*intergenic between NGK_RS11800 and NGK_RS11805	
	6817	0.203	NGK_RS01835	23S rRNA pseudouridine(1911/1915/1917) synthase RluD	
				**Tetracycline (TET)**	
	27095	0.470		*intergenic between NGK_RS07930 and NGK_RS07935	
	21468	0.205	NGK_RS06540	DUF3037 domain-containing protein	
[37926, 37927]	37927	0.196	*rpsJ*	30S ribosomal protein S10	✓
	29960	0.159		*intergenic between NGK_RS13555 and NGK_RS08865	
	37300	0.150	NGK_RS10900	methionyl-tRNA formyltransferase	
	40041	0.131	NGK_RS11710	TonB-dependent receptor	
	21467	0.121	NGK_RS06540	DUF3037 domain-containing protein	
	9785	0.120	NGK_RS02995	PBSX family phage terminase large subunit	
	9787	0.120	NGK_RS02995	PBSX family phage terminase large subunit	
	18761	0.119	NGK_RS05725	MULTISPECIES: Fe-S cluster assembly transcriptional regulator IscR	
				**Azithromycin (AZM)**	
	27421	0.424		*intergenic between NGK_RS13375 and NGK_RS07950	
	27690	0.420		*intergenic between NGK_RS08005 and NGK_RS08015	
	30659	0.300	NGK_RS09100	IS110 family transposase	
	36328	0.294	NGK_RS10580	pilus assembly protein	
	36810	0.290		*intergenic between NGK_RS10625 and NGK_RS10660	
	30434	0.278	NGK_RS08975	DUF1132 domain-containing protein	
	21513	0.269	NGK_RS06565	MULTISPECIES: hypothetical protein	
	39676	0.266	NGK_RS11620	homoserine kinase	
	36809	0.258		*intergenic between NGK_RS10625 and NGK_RS10660	
	29095	0.254	NGK_RS08360	MULTISPECIES: phosphatidylglycerophosphatase A	

The column "ID Range" lists the ranges of SNPs that fall in known AMR-associated genes (only) in our data. ID: ID of Identified SNP.
*NGK_RS01270: glutathione synthetase; NGK_RS01275: diacylglycerol kinase (DagK); NGK_RS09910: MULTISPECIES: HPr family phosphocarrier protein; NGK_RS09915: PTS sugar transporter subunit IIA; NGK_RS09815: iron uptake system protein EfeO; NGK_RS09830: murein transglycosylase; NGK_RS09405: competence protein ComE; NGK_RS09440: inner membrane protein YpjD; NGK_RS08825: competence protein ComE; NGK_RS13555: hypothetical protein, partial; NGK_RS11800: hemoglobin-haptoglobin-utilization protein; NGK_RS11805: DUF560 domain-containing protein; NGK_RS07930: lactoferrin/transferrin family TonB-dependent receptor; NGK_RS07935: transferrin-binding protein-like solute binding protein; NGK_RS08865: MULTISPECIES: P-II family nitrogen regulator; NGK_RS13375: hypothetical protein; NGK_RS07950: Fic family protein; NGK_RS08005: prephenate dehydratase; NGK_RS08015: membrane protein; NGK_RS10625: MULTISPECIES: RNA polymerase-binding protein DksA; NGK_RS10660: competence protein ComE.

Table 4 Numbers of SNPs identified by DNP-AAP, LASSO, and AdaBoost which occur in known AMR determinants listed in Table 2.

Drug \ Method	DNP-AAP	AdaBoost	LASSO
CIP	2	1	1
TET	1	1	1
PEN	2	1	1
CFX	1	1	2
AZM	1	0	0

Table 5 AUC for logistic regression classifiers built using the top SNPs identified by DNP-AAP, LASSO, and AdaBoost.

Drug \ Method	DNP-AAP	AdaBoost	LASSO
CIP	0.994	0.992	0.988
TET	0.969	0.921	0.852
PEN	0.974	0.995	0.962
CFX	0.976	0.959	0.952
AZM	0.949	0.974	0.961

Author details

[1]Department of Computer Science, University of Saskatchewan, 110 Science Place, S7N 5C9 Saskatoon, Canada. [2] Department of Animal & Poultry Science, University of Saskatchewan, 51 Campus Drive, S7N 5A8 Saskatoon, Canada. [3] Department of Mathematics and Statistics, University of Saskatchewan, 106 Wiggins Road, S7N 5E6 Saskatoon, Canada. [4] Department of Biochemistry, Microbiology and Immunology, University of Saskatchewan, 107 Wiggins Road, S7N 5E5 Saskatoon, Canada. [5] Vaccine and Infectious Disease Organization – International Vaccine Center, 120 Veterinary Rd, S7N 5E3 Saskatoon, Canada.

References

1. Perkins, S., Lacker, K., Theiler, J.: Grafting: Fast, incremental feature selection by gradient descent in function space. Journal of Machine Learning Research **3**, 1333–1356 (2003)
2. Liu, B., Wei, Y., Zhang, Y., Yang, Q.: Deep neural networks for high dimension, low sample size data. In: Sierra, C. (ed.) Proceedings of the 26th International Joint Conference on Artificial Intelligence: 19-25 August 2017; Melbourne, pp. 2287–2293 (2017)
3. Public Health Agency of Canada: National Surveillance of Antimicrobial Susceptibilities of *Neisseria Gonorrhoeae* Annual Summary 2014. http://healthycanadians.gc.ca/publications/drugs-products-medicaments-produits/2014-neisseria/alt/surveillance-gonorrhoeae-2014-eng.pdf
4. Centers for Disease Control and Prevention. https://www.cdc.gov/nchs/data/hus/hus07.pdf
5. World Health Organization: Global Action Plan to Control the Spread and Impact of Antimicrobial Resistance in *Neisseria Gonorrhoeae*. http://apps.who.int/iris/bitstream/10665/44863/1/9789241503501_eng.pdf
6. Harrison, O.B., Clemence, M., Dillard, J.P., Tang, C.M., Trees, D., Grad, Y.H., Maiden, M.C.J.: Genomic analyses of *Neisseria gonorrhoeae* reveal an association of the gonococcal genetic island with antimicrobial resistance. Journal of Infection **73**(6), 578–587 (2016)
7. Eyre, D.W., De Silva, D., Cole, K., Peters, J., Cole, M.J., Grad, Y.H., Demczuk, W., Martin, I., Mulvey, M.R., Crook, D.W., Walker, A.S., Peto, T.E.A., Paul, J.: WGS to predict antibiotic MICs for *Neisseria gonorrhoeae*. J Antimicrob Chemother **72**, 1937–1947 (2017)
8. Unemo, M., Shafer, W.M.: Genomic analyses of antimicrobial resistance in *Neisseria gonorrhoeae* in the 21st century: past, evolution, and future. Clinical Microbiology Reviews **27**(3), 587–613 (2014)
